# Dynamic QoS Management for a Flexible 5G/6G Network Core: A Step toward a Higher Programmability

**DOI:** 10.3390/s22082849

**Published:** 2022-04-07

**Authors:** Petar D. Bojović, Teodor Malbašić, Dušan Vujošević, Goran Martić, Živko Bojović

**Affiliations:** 1 The School of Computing, Union University Belgrade, 6/6 Knez Mihailova, 11000 Belgrade, Serbia; dvujosevic@raf.rs (D.V.); gmartic@icloud.com (G.M.); 2 RT-RK Institute for Computer Based Systems, 23a Narodnog Fronta, 21000 Novi Sad, Serbia; malbasicteodor@gmail.com; 3 Faculty of Technical Sciences, University of Novi Sad, 6 Trg Dositeja Obradovića, 21102 Novi Sad, Serbia; zivkocbojovic67@gmail.com

**Keywords:** 5G/6G, dynamic QoS management, network slicing, software-defined networking, queue management

## Abstract

The academic and professional community has recently started to develop the concept of 6G networks. The scientists have defined key performance indicators and pursued large-scale automation, ambient sensing intelligence, and pervasive artificial intelligence. They put great efforts into implementing new network access and edge computing solutions. However, further progress depends on developing a more flexible core infrastructure according to more complex QoS requirements. Our research aims to provide 5G/6G core flexibility by customizing and optimizing network slices and introducing a higher level of programmability. We bind similar services in a group, manage them as a single slice, and enable a higher level of programmability as a prerequisite for dynamic QoS. The current 5G solutions primarily use predefined queues, so we have developed highly flexible, dynamic queue management software and moved it entirely to the application layer (reducing dependence on the physical network infrastructure). Further, we have emulated a testbed environment as realistically as possible to verify the proposed model capabilities. Obtained results confirm the validity of the proposed dynamic QoS management model for configuring queues’ parameters according to the service management requirements. Moreover, the proposed solution can also be applied efficiently to 5G core networks to resolve complex service requirements.

## 1. Introduction

The development of advanced technologies, new communication types, and the rapid implementation of more intelligent and demanding services, are the leading trends in modern society. The 5G networks have significantly intensified the usage of software technologies, all virtualization techniques, massive MIMO, ultra-densification, and new frequency bands [1] to match these trends. A current research direction has been pursued toward full automation and far more comprehensive implementation of remote management systems. However, 5G still cannot provide satisfactory solutions for many services such as, e.g., multi-sensory holographic teleportation, which requires Tbps data rates and ms latencies. Moreover, it cannot push connectivity density well beyond the 106-km^2^ metric (an essential requirement for the upcoming Industry X.0 paradigm) [2]. With all this in mind, the scientific community has defined Key Performance Indicators (KPIs) for the 6G ecosystem [3]. These KPIs will be carefully measured for an efficient transition from a Network-as-an-Infrastructure (NaaI) to a Network-as-a-Service architecture (NaaS) [4]. Thus, it is clear that network cloudification intensifies with the critical role of networking in the latest cloud/edge computing technologies, which leads to a convergence of networking and cloud/edge computing [5].

A 6G network should feature a service-based End-to-End (E2E) architecture in which software-defined networking (SDN), network function virtualization (NFV), and network slicing play fundamental roles [6,7]. These technologies introduce significantly higher programmability into the network and allow the creation of multiple logical networks on a shared physical infrastructure. We expect that they provide higher data rates, enhanced spectral and energy efficiency, better coverage, wide bandwidths, extremely high reliability, ultra-low latency, and dynamic QoS management in all network segments. Dynamic QoS management is essential for achieving high flexibility in available network resource usage. However, in most SDN implementations, a required QoS provides statically, by resource reservation, according to the predefined rules in forwarding engines. Packets map into existing queues with different assigned priorities [8]. Therefore, a lack of dynamic queue management (i.e., the creation, modification, and removal of queues) is evident.

Our research focuses on defining and verifying an acceptable method for dynamic QoS. We propose a framework for the 5G/6G network core design, including new service management. It implies service binding into service groups that are most likely offered by the same service provider. Thus, each service group should be a separate slice controlled and managed by its authoritative controller. The dynamic queue management presented in the paper aims to provide dynamic QoS. The central part of the solution is configuring the queues’ parameters dynamically according to the requirements specified through the service management on the application layer. Concretely, we use the OVSDB protocol [9] to extend the SDN controller functionality in addition to the features supported by the OpenFlow protocol [10]. Overall, the proposed approach introduces a considerably higher degree of programmability to build a flexible infrastructure (both real and virtual). From the scientific and methodological point of view, it brings the following achievements:

- Optimized E2E slicing in shared networks across multiple domains;
- Efficient dynamic QoS management in the SDN environment—true dynamic queueing.

We have organized the rest of the paper as follows: Section 2 gives a background of core technology. Section 3 reviews the concept of shared networks (Section 3.1) and presents solutions for dynamic QoS management in SDN networks based on the existing literature (Section 3.2). Further, in Section 3.3, we explain the motivation for our study and compare our approach with state-of-the-art research in more detail. In Section 4, we present proposals for the design of multi-slice architecture (Section 4.1) and the methodology for dynamic QoS (Section 4.2). Section 5 describes the testbed design and the implementation of the proposed methodology and testing scenarios. The presentation of results and the performance evaluation are in Section 6. Finally, we conclude the paper with an overview of possible future work.

## 2. Background

The future 5G/6G networks will be hybrid systems, complex architectures encompassing wireless and optical communications. Most research focuses on implementing new technologies in access networks and edge computing to realize the ultra-high peak rate, ultra-mass access, and ultra-high reliability in a network. However, only introducing these new technologies is not enough to solve all the challenges within the current architecture. Improving the network’s core is necessary to achieve a robust, flexible, cost-effective, and intelligent network [11]. SDN and NFV technologies are viable enablers for providing such architecture and operations. The flexibility is achieved by separating network functions from hardware and implementing them into the software. This separation leads to high-level network programmability, enabling the necessary conditions for efficient implementation and shortening the time to market for new services [12]. The result is a rapid growth in the number of users, new types of communication, the emergence of more complex services, and the need to enable Over-The-Top (OTT) players to connect their services and applications to 5G/6G infrastructure [13].

We examine how to improve the QoS performances of an SDN network when the number of supported services increases and network resource utilization changes. The SDN controller has a crucial role in satisfying the requirements of different services much more flexibly and providing precise control of each flow. However, some functionalities that should provide a more flexible configuration in the SDN network are still missing. For example, the existing OpenFlow switches allow only the Match-Action packet processing method. This method uses a fixed set of fields and the limited specification of the OpenFlow protocol, with a limited set of packet processing actions [14]. Our proposal aims to fulfill specific dynamic QoS requirements through dynamic queue management.

## 3. Related Work

While diversifying QoS requirements, it is necessary to develop solutions that will enable us to customize and optimize network slices. Therefore, research in this area must be directed to find an optimal solution for slicing in shared networks and dynamic QoS management across SDN infrastructure (both real and virtual infrastructure).

### 3.1. Slicing in Shared 5G/6G Networks

Slices are composed of NFVs that are dynamically distributed across the network and span multiple administrative domains. NFV aims to decouple the software from the data plane, while SDN provides a centralized logic for the configuration and management of network infrastructure. For shared 5G/6G networks, a very high degree of multi-tenancy, where strong service guarantees force isolating resources across slices, is vital for the reliable operation of 5G/6G systems [15]. In a shared 5G/6G network, i.e., in a multi-tenant environment, network slicing enables different tenants (e.g., users, services, and vendors) to share the same infrastructure and to build fully decoupled E2E networks [3,16]. This environment enables economic benefits for providers since it decreases the need for investment in separate infrastructures.

Moreover, it decouples the role of the infrastructure provider from the service provider (tenant), creating a market environment that increases competition on both sides. Concerning security, the use of shared infrastructure implies the mandatory implementation of solutions that should enable maximum traffic isolation between different tenants, which a multi-slice organization achieves [17]. Due to the software-defined nature of slices, heterogeneous infrastructure resources can be leveraged in a streamlined and cost-effective manner [18,19]. The authors of [20] have proposed a mixed-integer linear program minimizing latency under the client’s demand and server’s bandwidth constraints based on low and medium traffic intensities. With these objectives, it turns out that concentrating the traffic on the closest server yields an optimal solution in the NFV context.

The deployment of intelligent and adaptive environments/applications is a driver for 5G/6G network evolution. In [21], the authors stress that the 6G network should provide lower latency and higher reliability, enabling ultra-massive M2M communications and the usage of low-power communications. The authors in [22] assume that the dynamic network configuration is based on current network utilization. By establishing efficient network sharing schemes, multiple tenants which may own conflicting resource requirements obtain access to the different parts of the limited resources.

The implementation process of intelligent services in 5G and 6G multiservice environments draws particular attention [23]. The authors devise a taxonomy for network slicing using different parameters (e.g., fundamental design principles, service function chaining schemes, physical infrastructures, and security) and present several requirements for network slicing and possible solutions.

A study in [24] analyzes recent trends and challenges in network slicing, the 3GPP standardization process, and many related mechanisms. However, certain shortcomings are noticeable, e.g., the complexity of the 3GPP solution, missing the tenant’s portal, and the separated orchestration of network slices and their services. In [25,26], the authors point out these limitations in current 5G network slicing implementations, especially management and orchestration. Moreover, they notice that slice-level operations are not well-separated from other processes, resulting in complex interactions between the 5G network components in the overall network slicing architecture.

The authors of [25] propose a modular 6G-LEGO framework in which slices have embedded (in-slice) management and orchestration support, and multi-domain slices rely on multiple slice-agnostic orchestrators. In [26], the authors advocate for what they call zero-touch 6G massive network slicing, introducing a novel analytical engine to perform slice-level resource prediction by learning offline while respecting some service-level agreement (SLA) constraints.

The authors in [27] consider network slicing at three different layers (the infrastructure, network, and service layer) and present an overview of the existing network slicing architectures. In [5,28], authors describe the main objective of the 5GEx project to employ a decentralized cascade approach for inter-provider service orchestration. Combining Network Function Infrastructure as a Service (NFVIaaS), Virtual Network Function as a Service (VNFaaS), and connectivity services, the 5GEx introduces a Slice-as-a-Service (SlaaS) paradigm. According to this approach, each provider acts as a service reseller to customers, and the delivered services may contain sub-services and/or resources from other providers.

A comprehensive overview of technologies and the fundamental principles of network slice architecture building [29] points out the requirements that need to be solved in core networks to realize network slicing and the challenges regarding slice resource allocation. The authors in [30] propose new technologies, such as air interface and transmission technologies and novel network architecture. They suggest network slicing to overcome the 5G shortcomings, together with cell-free architecture and cloud/fog/edge computing. One of the essential characteristics of a future network will be the extreme flexibility to build and configure more than 10 million private networks and many automated and advanced network slicing. In [31], the authors expect to see up to 10.000 slices in a service provider network that smartly shares virtual resources.

### 3.2. Dynamic QoS Management in SDN Environment

The academic and professional community has conducted a significant amount of research regarding QoS in SDN networks with the OpenFlow protocol in the previous period. The development of 5G and 6G network architecture drives the frequency of research on this topic, focusing on providing adaptive QoS in an SDN environment, given switch diversity, end-to-end quality of experience (QoE), and dynamic queue management [32].

In [33], the authors present a solution for providing end-to-end QoS. They implement a QoS controller which routes the traffic flows and allocates appropriate resources along the traffic path according to the required level of performance and guarantees that are needed for different applications. The focus lies on the QoS control plane aspects and not on the impact these mechanisms cause when applied dynamically.

Some authors emphasize switching diversity and SDN control plane operations in their research. In [34,35,36,37], the authors analyze switch scalability and performance regarding the increasing number of rules and traffic priorities. They also take into consideration the impact of some control plane anomalies.

The studies [38,39] examine the OpenFlow data rate-limit feature for a combination of “elephant” and “mice” traffic flows, which happen regularly in production networks. Research presented in [38] was performed on a static QoS setup in the Mininet environment, while, in [39], the authors used the Pica8 P3290 switch.

In [40], the authors leverage Deep Packet Inspection (DPI) to enable application-aware QoS mechanisms for end-to-end traffic engineering systems in SDN networks. The implemented system increases throughput and reduces latency by distributing flows to multiple queues with different priorities under the network administrator’s control. Moreover, the adaptive QoS mechanism can be helpful in the first phase of building an SDN environment in a hybrid SDN where OpenFlow and traditional networks exist together [41].

Some authors go in other directions and, in their research, pay special attention to challenges that arise in SDNs with instant services. Their research aims to develop a bandwidth management mechanism and to apply an appropriate rate limitation policy under traffic congestion for each application. For instance, they develop, e.g., a mechanism for dynamic routing in the SDN network based on real-time-monitored statistics and calculating optimal routes with minimum cost [42]. They integrate this mechanism with a specific machine learning algorithm to enable flow classification and application identification.

Link congestion is a severe problem that significantly affects a device’s performance and the entire network. In [43], the authors analyze this problem in the context of queuing delay and packet loss on routers. They propose the Weighted Queue Dynamic Active Queue Management (WQDAQM) based on dynamic monitoring and reaction, which defines queue weights and thresholds, dynamically adjusted based on traffic load.

Recently, authors have focused on integrating SDN architecture with the Service-Oriented Architecture (SOA) concept [44]. This architecture concept brings on the challenge of maintaining the QoS in networks. The authors conclude that gaps still exist in developing and applying QoS management in SOA-based SDNs. They categorize the QoS management into five main categories and analyze the impact of categories on guaranteeing end-to-end QoS provisioning.

Dynamic QoS management will become more and more significant with 5G and especially 6G mobile communications, and requires fast and efficient responses to many challenges such as lower delays, higher traffic volumes, and data rates. In [45], the authors propose solutions within the synergy of technologies, such as NFV and SDN techniques, over the cloud-enabled radio systems. They point to potential benefits such as enabling resource pooling, scalability, layered interworking, and spectral efficiency.

From the aspect of large or carrier-grade networks based on SDN technology such as 5G and 6G networks, specific components of the OpenFlow architecture will be subject to continuous development. Changes within the OpenFlow standards, which relate to QoS, represent a considerable challenge because, within the OpenFlow protocol, there is still much free space for implementing new, more comprehensive, and feature-rich solutions. In order to perform QoS experiments, the authors in [46] introduce an architectural extension for the OpenFlow environment. Further, the authors identify current QoS limitations in OpenFlow switches, propose and implement enhancements related to queue configuration, and extend the OpenFlow protocol by defining queue settings based on specific queue types. Some authors have focused on management and control plane aspects to provide centralized end-to-end QoS [47,48]. Without extending the OpenFlow specification, they have opted to implement a QoS policy management framework that provides an interface for specifying QoS-based SLAs and enforces the use of the OpenFlow API.

Using GI/M/1/K and M/M/1 queues from the queuing theory, the authors in [49] model switches and a controller. They compare two different switch implementations (a shared buffer and two priority buffers) and conclude that priority buffering should be the preferred mechanism for mobile networks.

### 3.3. Motivation

We can conclude that most research focuses on access networks and edge computing. Keeping in mind the heterogeneity of future networks, we point out that it is necessary to consider the potential challenges in the core of future networks in detail. To achieve the fundamental goals of 5G/6G, we need to foster higher system capacity, higher data rate, lower latency, and improve the Quality of Service (QoS). There are studies that deal with the implementation of dynamic QoS in 5G/6G networks. However, they do not deal with the issue of dynamic queue management (i.e., the creation of queues and their modification and removal).

Dynamic queue management should significantly provide the necessary flexibility in core networks. Relying solely upon predefined queues, none of the works representing the state of the art of existing research has foreseen the possibility of the dynamic creation of QoS queues [50]. Regarding QoS, such an approach is a limiting factor in system flexibility. Our focus is, thus, to implement a solution that can dynamically ensure the Quality of Service (QoS) in SDN as a core of networks.

Flexibility and scalability should go hand in hand [51], and we believe that network slicing combined with dynamic queue management could address the diverse service requirements. In that sense, we intend to apply an appropriate solution to provide a better slice organization within the existing multi-slice environment. Our idea of slice organization is to deploy a group of similar services as a single slice. Such a slice can consist of different providers’ services belonging to the same service group.

This approach would significantly benefit the network operator and the users since it would eliminate several limiting factors. Namely, the operator expands and enriches its services by allowing different providers to offer the same service group. In this way, users also benefit from the opportunity to choose a provider. The deployment of such a solution within the 5G/6G network implicitly encourages competition and puts user experience firmly in the foreground.

## 4. Architecture Design and Methodology

This section will first describe the proposed design of multislice network architecture. Further, we will present our proposal for QoS methodology based on dynamic queue management.

### 4.1. Design of Multi-Slice Architecture

The deployment of sliced network architecture should improve the network interoperability of wireless infrastructure providers to respond effectively to user requests, and it is closely related to providing a better QoE through adequate QoS. The previous research mainly uses existing queues or other reserved resources in the forwarding hardware, contrary to the SDN paradigm, whose primary goal is to increase network programmability. Considering this, we have started from the facts that, currently:

- No SDN controller enables standardized management of queues (other than flow assignment to queues);
- OpenFlow and OVSDB represent established standard protocols.

**Figure 1:**
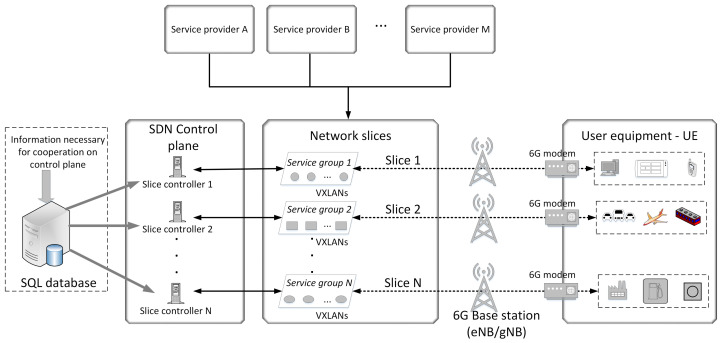
The proposed model of 5G/6G multi-slice environment.

In a multi-slice wireless environment, we need to define slice organization, which carries a challenge related to management complexity in future networks (Figure 1). Firstly, we need to bind related services that the same service provider most likely offers. It is reasonable to avoid using unnecessary resources to create a separate slice for each service. We believe that grouping services can ensure efficient real-time resource sharing according to business processes. Each group represents a separate slice controlled by its authoritative controller. To clarify the role and importance of service bonding in multi-slice 5G/6G networks, we have shown a use case applied in an intelligent vehicle setup (Appendix A, Table A1).

We can expect different providers to offer the same groups of services in the market. Our concept supports the possibility of providing a group of services from different providers within a single slice. We isolate services and service providers by leveraging existing network segmentation mechanisms (e.g., VLAN, VXLAN). This solution allows for a high level of scalability since the number of slices is not limited. We propose using VXLANs to technically separate providers that offer the same services group (within each slice, there can be 224 different providers appertaining to a single service group). This approach enables users to access different slices and choose technical and cost-effective offers, which significantly benefits users. Users have the opportunity to choose a service provider for each service group.

**Figure 2:**
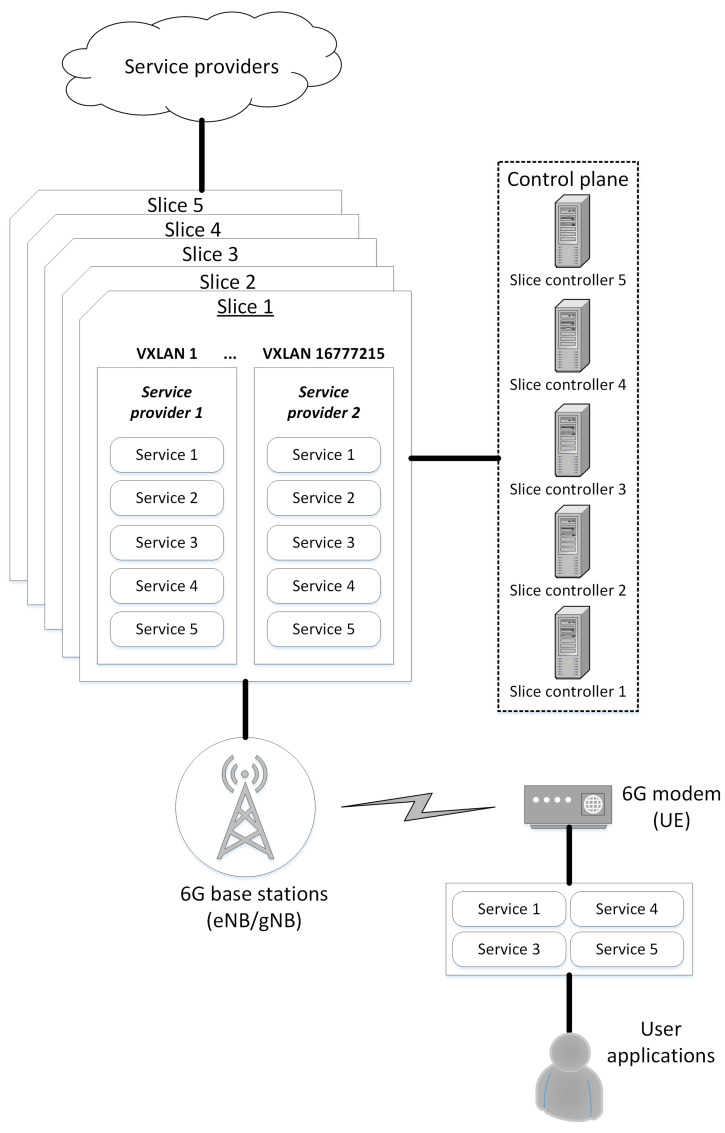
Service bonding and provider isolation in a multi-slice environment.

Multi-slice represents the E2E concept implemented in all network segments (RAN, Edge, Core, and transport) [26]. The SDN functionality of the 5G/6G network is available from the edge network to the 6G modem on the customer side. This infrastructure concept allows for fully open access to the service market, which benefits both clients and service providers (Figure 2).

### 4.2. Dynamic QoS Methodology

Knowing that it is necessary to establish comprehensive dynamic service quality control within each slice, we imply that a single SDN controller controls one slice in our solution. We accomplish the synchronization of slice controllers by utilizing a shared database. This database presents a well-managed repository containing information about network functions, resources, service providers, architecture, and orchestration processes. This information supports scheduling similar services in a service group for a specific network slice. We propose a dynamic QoS traffic management methodology in which we utilize dynamic queue management and apply mechanisms to make intelligent decisions for max rates of service groups.

#### 4.2.1. Dynamic Queues Management

The resource reservation is provisioned by effectively creating upload and download queues, thus providing the bandwidth requirements for that group of services. If requirements change and the user cancels the subscription to a specific group of services, the reservation of resources for those services should be unprovisioned by removing the upload and download queues. Automatically, the other groups of services (slices) can consume the released leftover bandwidth. Due to user mobility, we expect that variations in signal quality and strength between users and base stations will cause variability in total available bandwidth. In addition, the current congestion of a particular base station is a factor that can lead to a decrease in bandwidth per user. Thus, it is imperative to implement a mechanism that dynamically manages the QoS configuration of slices sensitive to packet loss, and we should treat them differently.

Moreover, by introducing dynamism into the QoS configuration, the following strategy can be applied—some less-critical services (e.g., Internet service) can be temporarily slowed down or even suspended until the link bandwidth capacity improves. In this paper, we did not evaluate the bandwidth capacity of a wireless link. We assume that we could predict available bandwidth from transmission parameters (e.g., signal strength, signal-to-noise ratio).

We propose a method for dynamic QoS through dynamic queue management that implies the ability to dynamically change queues’ parameter configurations according to the requirements specified through the slice orchestration on the application layer. To achieve this goal, we propose using the OVSDB protocol to extend the SDN controller functionality in addition to the features supported by the OpenFlow protocol. Namely, by applying the OVSDB protocol, we perform dynamic queue management (e.g., creating, deleting, and changing queue parameters dynamically).

**Figure 3:**
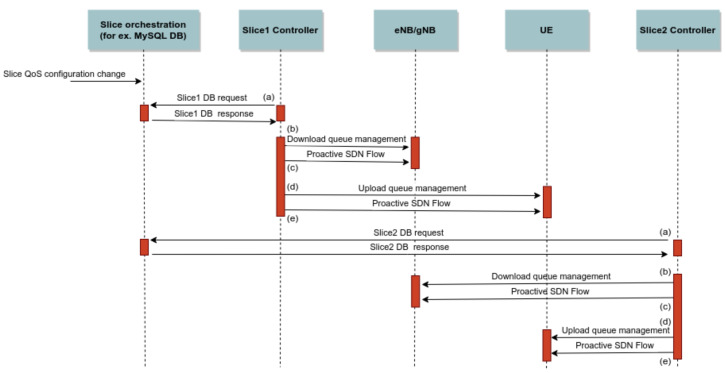
QoS policy change flow diagram.

We enable the dynamic control of link bandwidth by following the SDN paradigm through a combination of functionalities contained in the OpenFlow (queue flow assignment) and OVSDB protocols. For QoS to be bi-directionally regulated, queues should be set up on both SDN switches at the base station side (for downstream) and the user side (for upstream). Our proposal for dynamic QoS management is shown in the flow diagram in Figure 3.

The process depicted in Figure 3 begins with a request for a change of QoS policy for a specific service group in the slice orchestration database. This process contains five steps that each slice controller runs independently from other controllers:

(a) The slice controller retrieves the slice QoS policy for the slice it is in charge of, and if there are any changes in comparison with the previous state, it executes these changes;
(b) The slice controller sends a command to modify the queue to the base station via the OVSDB protocol;
(c) The slice controller creates a proactive flow to assign slice traffic to the appropriate queue on a base station;
(d) The slice controller sends a command to modify the queue to the UE via the OVSDB protocol;
(e) The slice controller creates a proactive flow to assign slice traffic to the appropriate queue on a UE.

#### 4.2.2. Bandwidth Allocation Mechanism

Dynamic queues’ management may not be sufficient to combat future challenges in high-demand networks. A higher level of intelligence is required to observe both service requirements and link capabilities before making the right decisions for max-rates and queue sizes. Therefore, we propose a link bandwidth allocation mechanism that uses information stored in the database and calculates max-rates for each user’s download and upload directions and slices. For each user *i* and slice *j*, the queue size (max-rate) is calculated using Formula (1):

(1) $$q_{ij}\;=\;\left\{\;\begin{array}{c} c_{i}w_{ij},\;\;\;\;t_{ij}\;\leq \;c_{i}w_{ij}\;\wedge \;s_{ij}\;+\;c_{i}w_{ij}\;\leq \;c_{i}\\ m_{ij},\;\;\;\;\;t_{ij}\;>\;c_{i}w_{ij}\;\wedge \;s_{ij}\;+\;m_{ij}\;\leq \;c_{i}\\ c_{i}\;-\;s_{ij},\;\begin{array}{c} \left(t_{ij}\;\leq \;c_{i}w_{ij}\;\wedge \;s_{ij}\;+\;c_{i}w_{ij}\;>\;c_{i}\right)\\ \vee \;(t_{ij}\;>\;c_{i}w_{ij}\;\wedge \;s_{ij}\;+\;m_{ij}\;>\;c_{i}) \end{array} \end{array}\right.$$

where $t_{ij}$ is the minimal download/upload rate for user *i* and slice *j*; $c_{i}$ is the download/upload capacity of the link for user *i*; $w_{ij}$ is the link capacity portion (percentage); $p_{ij}$ is the priority of slice *i*, user *j*; finally, $m_{ij}$ and $s_{ij}$ are calculated according to Formulas (2) and (3).

(2) $$m_{ij}\;=\;\min\left\{t_{ij},\;c_{i}\;-\;\sum_{k,l}t_{kl}\;|\;p_{kl}\;<\;p_{ij}\right\}$$

(3) $$s_{ij}\;=\;\sum_{k,l}\max\left\{c_{k}w_{kl},\;t_{kl}\right\}\;|\;p_{kl}\;<\;p_{ij}$$

According to Formula (1), we calculate queue size based on three criteria:

- Link capacity is sufficient so that all slice requirements can be satisfied;
- Proportional (percentile) bandwidth allocation is less than the minimum rate required by the slice (2);
- Slice requirement could not be satisfied due to reduced link capacity and bandwidth allocation by higher-priority slices (3).

In cases when the proportional allocation is less than the minimum rate, a minimum required rate is used. However, if higher-priority slices have already allocated bandwidth by the first two criteria, the slice could only use the remaining unallocated rate.

We calculate the link bandwidth allocation in descending order of priority. Therefore, we allocate bandwidth for slices with lower priority before those with a higher priority number.

## 5. Testbed Design

The next step is to design an adequate testbed environment according to the proposed methodology and evaluate customized slicing performance. After that, we will verify the benefits of dynamic QoS management. Our goal is to implement a proposed method of dynamic management to be pure and simple, limited to what we want to investigate. So, we will not address different queue scheduling techniques (e.g., WFQ, LLQ, and PQ), congestion mitigation techniques (e.g., via RED, WRED mechanisms), nor different queue policers (e.g., OVS ingress/egress and trtcm). Our solution leverages Open vSwitch Queues with the Linux Hierarchy Token Bucket (Linux-HTB) [52] and the limitation of max-rate queues. The following subsections describe the design of the testbed environment, how we have implemented the proposed methodology, the way of generating traffic, and testing scenarios to verify the methodology.

### 5.1. Testbed Setup

**Figure 4:**
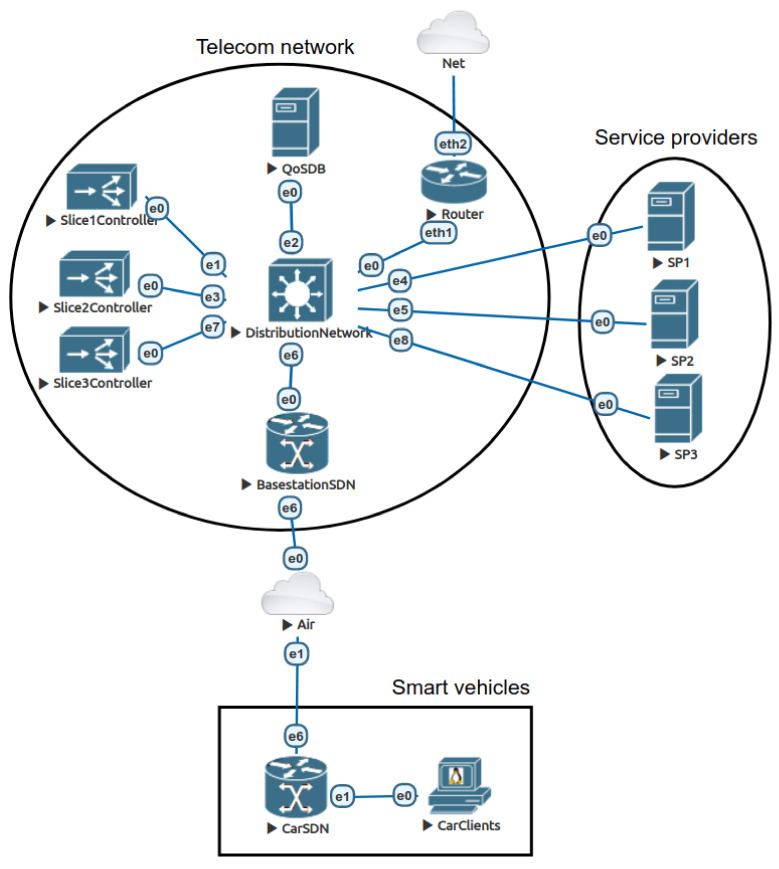
The design of the testbed environment.

In order to test and verify the capabilities of the proposed solution, it is necessary to implement an appropriate multi-slice environment that would adequately reflect the environment of 6G networks. Therefore, we have set up a virtual environment using the EVE-NG platform [53] to represent a simplified multi-slice environment for future networks. As shown in Figure 4, the design of the testbed environment consists of three parts:

- The 6G provider’s infrastructure consists of core and distribution layer devices and control plan management devices of its SDN network (SDN controllers and SDN base stations);
- Users (e.g., smart vehicles) access the 5G/6G infrastructure through a base station;
- Connections with third-party service providers.

We will verify the proposed methodology by using three independent slices. For testbed simplicity, we limit our service groups to one service per slice. Each slice is in charge of one service group (Table A1 in Appendix A). It is important to emphasize that we dedicate a separate SDN controller for managing each slice or a particular group of services. Moreover, to perform a more realistic testing environment, we arrange that one user obtains services from different providers. Table 1 presents the slice arrangement with each service group’s associated services and bandwidth requirements.

**Figure A1:**
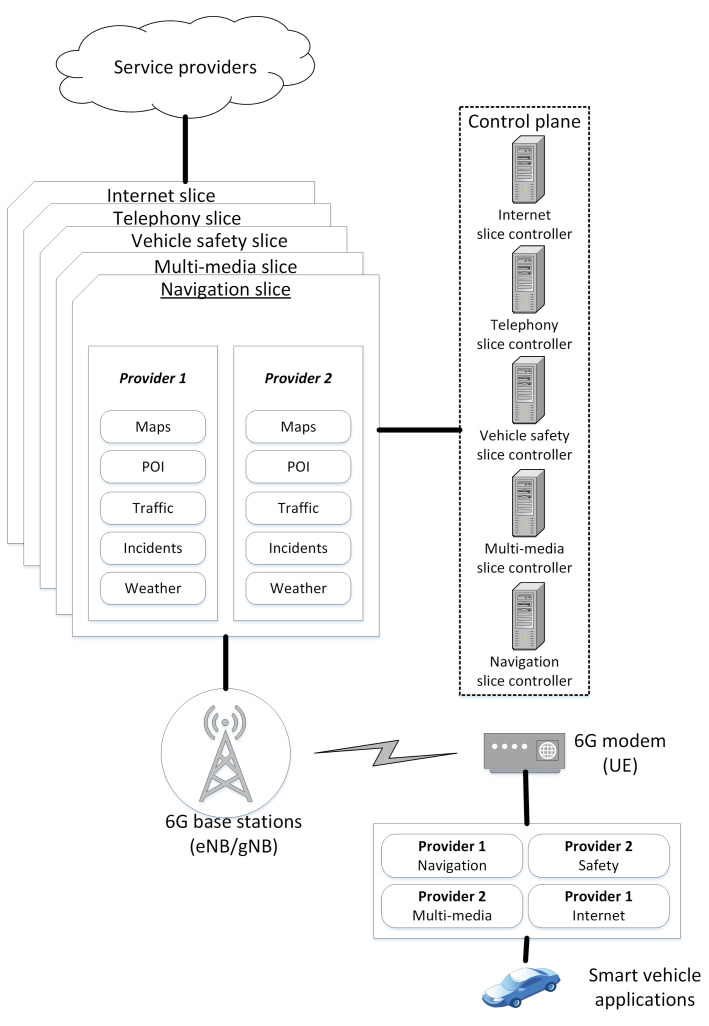
Example of smart vehicle multi-slice environment.

For each slice, we implement two components on the smart vehicle’s side. The first is the CarSDN component (an SDN switch, an integrated part of a 5G/6G modem) that provides user-side programmability, multi-slicing segmentation, and intelligent queue and traffic management. Applying the dynamic creation of queues and the ability to change their parameters enables the automated management of QoS mechanisms. Multi-slice segmentation is provided up to the CarSDN component (5G/6G modem). The example of practical use is presented in Appendix A, Figure A1. One of the roles of this component is to optimally manage the QoS of outgoing communications and to allow dynamic allocation of available upstream bandwidth. The client applications are the second component. They are embedded into the car’s ECUs (Electronic Control Unit) or user’s terminal equipment within the car.

As depicted in Figure 4, we have placed another manageable device between the SDN-aware station (BasestationSDN) and the car (CarSDN). This device has the role of introducing bandwidth availability limitations and, thus, enabling congestion into the virtual system (the congestion represents variable air transmission capability).

We include a separate service management server (QoSDB) in the testbed environment to enable dynamic changes of the QoS parameters and to provide necessary synchronization between slice controllers. This server contains an SQL database that specifies the configuration parameters of the desired QoS policies. The SDN controller in charge of a specific slice fetches the QoS policy from the SQL database and provisions the queues.

### 5.2. Methodology Implementation

**Figure 5:**
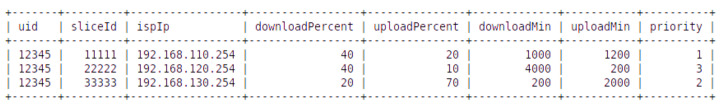
SQL database sample.

The implementation of the proposed methodology has started by analyzing QoS policies in an SQL database, which we use as a source of information required for dynamically configuring the QoS. Each entry in the database represents a group of services provided by a single slice and contains the following attributes (Figure 5):

- Unique user identification (e.g., IMSI and IMEI);
- The unique slice identifier;
- The IP address of the service group provider;
- Download link capacity portion (percentage) requirement at the slice level;
- Upload link capacity portion (percentage) requirement at the slice level;
- Minimum download bandwidth requirement at the slice level;
- Minimum upload bandwidth requirement at the slice level;
- Priority index to set bandwidth allocation order.

Additionally, our solution includes a logically separated SQL database table that stores the predicted total download and upload link capacity for each user. These stored values are used in the intelligent calculation of max bandwidth for every slice. We use these attributes to manage queues on the BasestationSDN and CarSDN switches. The slice ID is simply used to look up the other attributes from the SQL database. These slice policy attributes are then used to calculate download rate (maximum allowed download throughput of the queue) and set on BasestationSDN, while upload rate (maximum allowed upload throughput of the queue) is set on the CarSDN switch. The IP address of the service group’s server is used to define the proactive flows to which the corresponding queues will be applied. The software solution for dynamic QoS is written in Python and provided as an open-source project [53]. The responsibility of this software is to configure queue and flow tables on SDN switches according to database entries. Synchronization between the desired state (defined in SQL database) and an actual state on switches (queue and flow tables) is achieved periodically executing Python script. We initiate a single instance of the Python script at each SDN controller. Each SDN controller is responsible for its slice, and it is also responsible for both SDN switches when it comes to their services. Therefore, the purpose of the Python script is to handle the dynamic part of the QoS configuration on SDN switches. The Python script accepts two input parameters:

- SliceID;
- A period after which the script program repeats (in seconds)—this represents a database polling interval.

**Figure 6:**
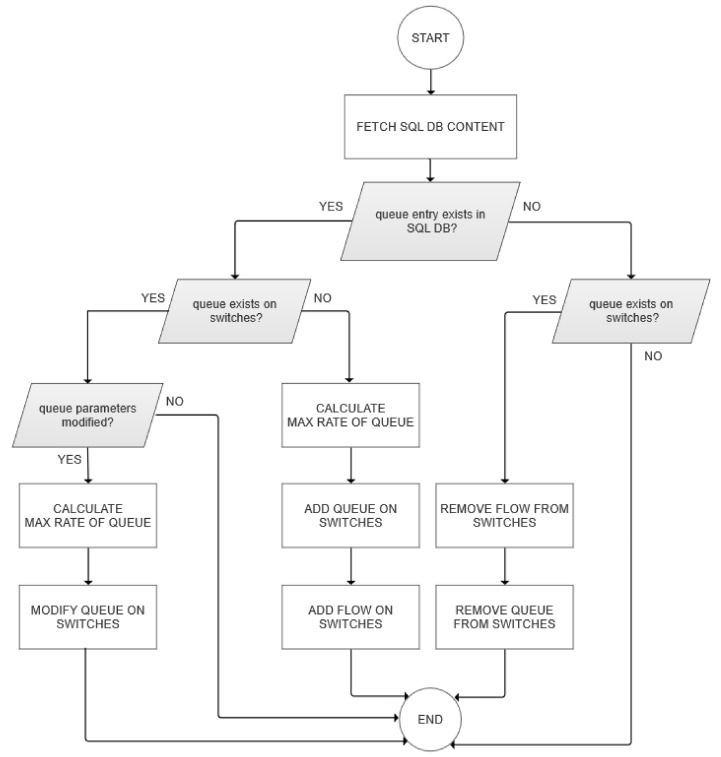
Process diagram of dynamic QoS management.

Queues and proactive flows are configured using the ovs-vsctl command line (part of the Open vSwitch tools). The implementation of the proposed methodology is described in Algorithm 1 and the process diagram is depicted in Figure 6.
**Algorithm 1** Dynamic QoS Algorithm.   **Input:** current queue query in SQL table1:**if** queue entry does not exist in SQL table **then**2:    **if** queue entry does not exist on switches **then**3:        do nothing4:    **else**5:        delete proactive flows on switches6:        delete queues on switches7:    **end if**8:**else**9:    **if** queue entry does not exist on switches **then**10:        add queues on switches11:        add proactive flows on switches12:    **else**13:        **if** current query result ≠ previous query result **then**14:           modify queues on switches15:        **end if**16:    **end if**17:**end if**

The first step is to fetch the content of the SQL database. Any change of download or upload parameters will result in the configuration of these queues that we apply on both switches. If queues have not been configured for the slice, we must create a new queue with its maximum throughput parameter for each switch. We also add the proactive flows supporting these new queues to the switches. In some cases, a specified queue entry does not exist in the SQL database, but its configuration exists on switches. The script removes both the queues and the flows from the switches. As already mentioned, the program repeats itself, while the second parameter of the script defines the period of repetition. The time interval for refreshing the QoS policy depends on the dynamic QoS implementation policy. Thus, for example, QoS policies do not need to be updated frequently for subscriptions and unsubscriptions to service groups. On the other hand, when it is necessary to provide a quick response to a changed state of link quality, it is necessary to support a fast update of QoS parameters. Our testbed presents a case when a quick QoS policy change is unnecessary, so we refresh every second. It could have contained different periods of updating QoS dynamics.

### 5.3. Traffic Generators

Instead of simulation, we decided to make an emulation of the environment and manage traffic by implementing the appropriate software on the servers to provide an adequate testbed environment. To better organize the service architecture, we have segmented service groups by creating different slices and arranging traffic with Table 2 parameters.

We have installed the Apache web service and PHP engine on server SP1 to generate traffic for the Internet (NET) slice. Then, we have created a PHP script that generates a random size response between 0.05 and 5 megabytes for each web request [54] generated once every second. Such a generator realistically reflects the Internet traffic and users’ needs for web surfing and downloading web resources of different sizes. Since users mainly use the HTTP protocol, web services rely on the TCP, which allows them to use built-in mechanisms to control data flows and even dynamically adapt to the current availability of the transport link bandwidth (through the windowing mechanism).

We have implemented two more slices (service groups). These two slices are named the Multimedia and Security slice. The Multimedia (MM) slice relies on multicast traffic or Video-on-Demand (VoD) video signal transmission. They use UDP streaming and do not have automatic flow-control mechanisms. We have used the D-ITG tool [55] to generate traffic with an uneven exponential distribution. In the direction from the service provider to the user, we have generated around 2000 packets per second with a total throughput between 5.5–6.2 Mbps.

The Security slice (SEC) has the role of performing constant video streaming of surveillance signals and sensor values to the “black box” cloud. The purpose of these services is to allow real-time analysis and enable the forensics of adverse events and optimize the operation of all engines within the vehicle. In the direction from the vehicle to the service provider, we have generated traffic of around 900 packets per second and a total throughput of 2.8–3.2 Mbps. Similar to the MM slice, we have used the D-ITG tool to generate an exponential traffic distribution.

### 5.4. Testing Scenarios

To evaluate the proposed solution for dynamic QoS in a multi-slice network, we have defined different testing scenarios and their parameters (Table 3):

- Scenario 1—represents a testing baseline with enough bandwidth capacity for two active service groups. An SQL database consists of only two QoS policies that slice controllers use to create instructions to manage specific SDN components;
- Scenario 2—introduces a third slice that requires the dynamic creation of queues and the redistribution of total link capacity with still enough bandwidth for all slice requirements;
- Scenario 3—introduces congestion in the testbed environment by limiting the link bandwidth that service groups share; thus, we initiate bandwidth reallocation and test the proposed methodology;
- Scenario 4—applies different QoS policies showing the dynamic nature of policy adjustment in a reduced-capacity setup;
- Scenario 5—aims to show the solution’s behavior when needed to perform dynamic release of service group resources. The resource release is necessary if the service group is no longer active, and the dynamic reconfiguration of free resources can be performed for active service groups.

The testing process is automated so that each test scenario is active for 60 s. After that, we switch to the next scenario.

Recording the performance of the set testbed was performed using the following tools:

- *bwm-ng* tool—used to monitor and log the active throughput of physical interfaces [56] on the CarClient site (for download-monitoring purposes) and the DistributionNetwork switch ( for upload-monitoring purposes);
- *D-ITG* tool [51]—used for logging and analyzing QoS parameters (delay, jitter, and packet loss) for UDP streaming on receiver pages on CarClient and SP3 server;
- *tcpdump* tool—used to collect complete traffic logs in the testing process on the DistributionNetwork switch.

For each slice’s SDN controller, we initiate one instance of a developed script for dynamic QoS management (Section 4.2), which only monitors the SQL table for one service group (slice ID). If using multiple services in one slice, or multiple vendors of the same service, it is necessary to run multiple instances with a different SliceID. In our test case, these instances have been initiated with an SQL pool interval of every second to achieve a faster response.

## 6. Results and Evaluation

The testing process aims to evaluate resource utilization of the proposed dynamic QoS management method in a realistic environment. As a proof of concept, we perform multiple cycles to verify the correct operation of each aspect of the proposed solution.

Applying database QoS policies (Section 4.2.2) on the proposed testing scenarios, we obtained Table 4. The implemented solution calculated Download and Upload max-rates for each slice and scenario by following the proposed allocation procedure. The majority of max-rate values were allocated using the first criteria in (1). In Scenarios 3 and 4, where link capacity is reduced below slice requirements, we can observe the use of second and third (bolded and underlined in Table 4, respectively) allocation criteria from (1)). Calculated max-rates are then dynamically applied to the appropriate SDN in queue modification.

**Figure 7:**
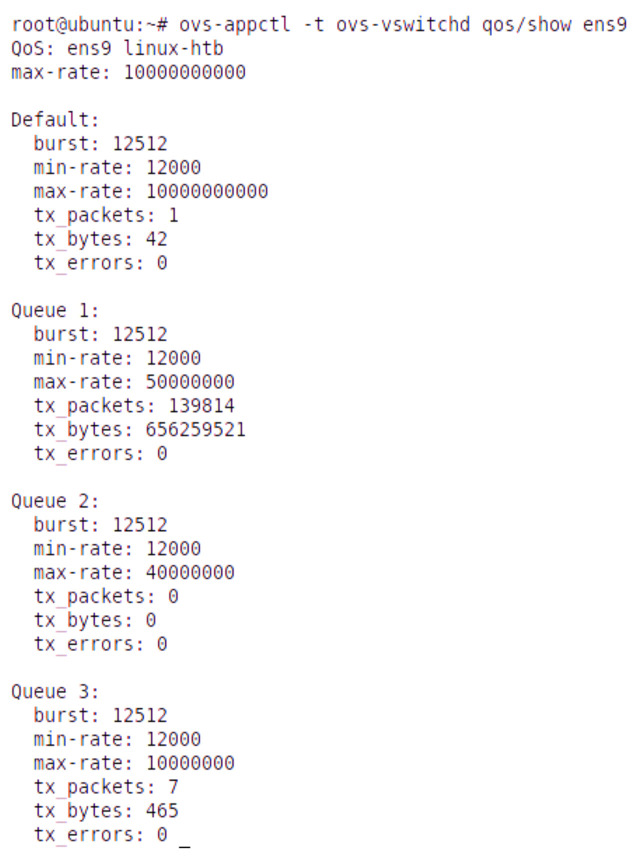
Base station OVS queue parameters in Scenario 2.

Figure 7 shows the base station queue parameters in Scenario 2. It also shows the max queue size (max-rate), min queue size (min-rate), burst rate, and statistics for transferred packets, bytes, and errors for each slice.

**Figure 8:**
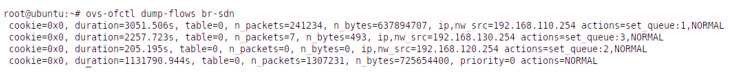
Base station flow table in Scenario 2.

Further, on Figure 8, we show the flow table content, with proactive flows on base station OVS in Scenario 2.

During this process, we perform multiple cycles. We have noticed consistent results regarding the measured performance parameters, and the only observed variance is caused by the random size of the WEB service response.

**Figure 9:**
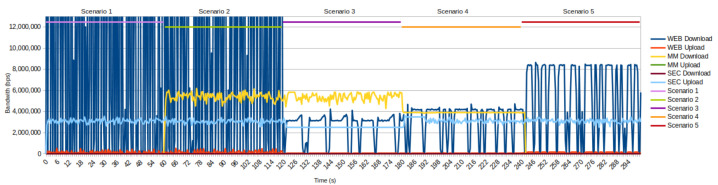
Bandwidth usage during testing process.

In Figure 9, we present test results, which show the bandwidth utilization in different test scenarios.

The first scenario represents a baseline setup where all service groups have the required bandwidth. The WEB service takes up to 40 Mbps, so every transaction executes in less than one second. The SEC upload streaming service also demonstrates its behavior under baseline conditions.

In Scenario 2, we introduce a new service group (in our case, MM service) by defining a new SQL record. After Scenario 2 starts, the SDN controller in charge of the observed slice initiates the creation of new queues for that service group. The new MM service does not suffer from performance degradation because there is still enough shared bandwidth for all traffic.

Scenario 3 introduces bandwidth reduction on the shared link, which causes congestion in both directions (upload and download). In the download direction, the link has a maximum bandwidth of 10 Mbps, while the WEB and MM services together require around 47 Mbps of bandwidth for uninterrupted operation. The problem of congestion can be immediately noticed by analyzing the responses of WEB requests because large-size responses can not finish in less than one second. Instead, the packet transmission must be redistributed over time. The available bandwidth has been redistributed according to the policies in the SQL table and the proposed bandwidth allocation mechanism. We allocate a bandwidth of 1 Mbps for the SEC service, 6 Mbps for the MM service, and 3 Mbps for the WEB service. SEC and MM services have higher priority than the WEB service. Therefore, the WEB service receives the remaining (available) bandwidth (less than required by the prescribed policy). According to the policy, the only visible change in upload direction is SEC service bandwidth reduction.

The results obtained by testing in Scenario 4 indicate the effects of a dynamic change in QoS policy. The dynamic change of SQL policy has led to a change in service priorities, and the WEB service obtains the highest priority in resource allocation. In the download direction for the WEB and MM services, we allocate 4 Mbps, and we allocate 2 Mbps to the SEC service, following QoS policies. However, bandwidth allocation was completed according to service priorities in the upload direction. A total of 1.2 Mbps has been allocated for the WEB service, which has the highest priority, while 3.5 Mbps has been allocated for the SEC service. The remaining bandwidth is allocated to the MM service (less than defined in the QoS policy).

Scenario 5 shows a situation in which one of the services is no longer in use, so it is possible to perform a dynamic reallocation of freed resources to give active service groups a higher bandwidth and, thus, improve the services’ performances. In our example (the bandwidth previously allocated by the QoS policy for the MM service), we can now reallocate for remaining services, which immediately shows the increased number of transactions. This scenario shows that it is possible to delete queues for service groups that are no longer needed.

**Figure 10:**
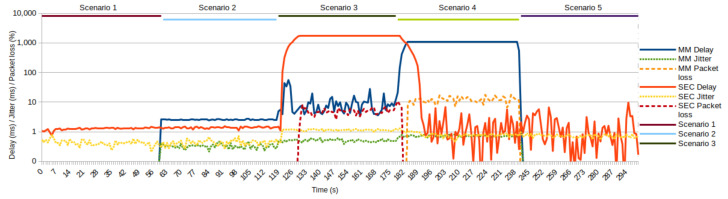
QoS parameters during testing.

Reducing bandwidth below the level required by the services leads to a reduction in the performance of that service. Figure 10 shows the impact of congestion and the application of QoS policies on the performance of individual service groups.

As we can observe, Figure 10 clearly shows that the limitation of the SEC service group upload in Scenario 3 introduces significant delay and packet drops. The service group requires up to 3.2 Mbps (Table 1), but the QoS policy allocates 2.5 Mbps. Therefore, when the queues are filled, all future packages from this service group are dropped. A similar case is with the MM service, where traffic is generated with exponential distribution up to 6.2 Mbps, but the download link is only 6 Mbps.

Further, Scenario 4 introduces even more reduction in the bandwidth of the MM service group (4 Mbps for download link), which results in a considerable delay and packet drops. However, this is an indicator of poor service performance for this streaming service. However, even application services that use UDP streaming often have some mechanisms for dynamic reduction in transmission throughput. For example, video streaming services use the possibility of a dynamic bitrate. When the client notices the link degradation and packet loss, it can ask the server to reduce bitrate (i.e., reduce the quality of the video stream until the packet loss is eliminated). Our testing scenario did not include such a bitrate reduction concept. We can notice that the SEC service group in Scenarios 4 and 5 did not recover the congestion introduced in Scenario 3.

## 7. Conclusions

Network slicing has an essential role in research aiming to develop new concepts of flexible network architecture. The current level of maturity and the constant development and improvement of this technology point out that network slicing will soon become a network standard and play a vital role in future network designs and operations. Therefore, developing a more efficient concept of a multi-slice organization is crucial. This paper proposes an efficient model that enables customized slicing in future network architectures. The basis of this model is slice-level architecture and dynamic QoS management.

We propose a solution that implies similar services binding in a group and managing them as a single slice. Further, we join providers that offer the same services to a single slice; this way we can specialize a network slice for specific purposes, thus providing a certain level of flexibility to the network core architecture and pointing to the potential of this architecture benefits for the network operator and its customer. Such well-organized and managed slices support multiple service providers isolated in each slice using some of the available segmentation technologies (e.g., VXLAN, VLAN).

According to the literature review presented in Section 3, it is evident that current research primarily uses a predefined queue and lacks fully dynamic queue management. Such an approach is contrary to the SDN paradigm, which implies introducing more and more programmability into a network. This represents a limiting parameter for the flexibility of the future core network. With this in mind, in the paper, we propose a new solution that eliminates this limitation and applies high-level programmability in QoS management. Each slice includes infrastructure devices and a separate SDN controller that controls this slice. Implementing highly flexible software solutions can significantly reduce the required knowledge of the physical network infrastructure. Such a solution can enable the customization of routes in network slices based on adequate QoS policy, representing an additional degree of flexibility in the 5G/6G infrastructure. In this manner, we move the QoS policies management entirely to the application layer. The presented methodology includes a bandwidth allocation mechanism by applying complex QoS policies (multiparameter policy). We wish to notice that, in a production environment, the dynamics of switching among scenarios would be managed by mechanisms of decision making based on current traffic, which are out of the scope of this testbed.

This paper does not address how QoS policies should look in future networks because this is still a very complex topic. We have proposed mechanisms to dynamically implement any planned QoS policy by allowing flexible and dynamic network parameters based on SQL DB service requirements. The proposed mechanism can offer only the best effort to satisfy all those requirements.

The future work will include the design of QoS policies in a multi-slice environment. Moreover, we will research the influence that other QoS mechanisms, such as different schedulers and traffic shapers, have on overall multi-slice QoS. In future research, we will focus on the connection of network slicing with RAN (Radio Access Network), and on how to enable a shared RAN for network slicing to accommodate highly diverse services.

## Figures and Tables

**Table 1:** Slice arrangement in testbed.

Slice Name	Service Group	Download Throughput	Upload Throughput
Internet	Web surfing	0.5–40 Mbps, 150–1500 pps	0–0.4 Mbps, 50–300 pps
Multimedia	Video streaming	5.5–6.2 Mbps, 1800–2200 pps	0–0.2 Mbps, 0–20 pps
Security	Sensors and surveillance stream	0–0.2 Mbps, 0–20 pps	2.8–3.2 Mbps, 820–940 pps

**Table 2:** Slice traffic parameters.

Slice	SliceID	Traffic Type	Evaluation Tool
Internet (NET)	11111	HTTP TCP	Apache/PHP script [54]
Multimedia (MM)	22222	UDP Stream	D-ITG [55]
Security (SEC)	33333	UDP Stream	D-ITG

**Table 3:** Scenario parameters.

	Scenario 1			Scenario 2			Scenario 3			Scenario 4			Scenario 5		
Slice	WEB	MM	SEC	WEB	MM	SEC	WEB	MM	SEC	WEB	MM	SEC	WEB	MM	SEC
Service groups running	*√*	×	*√*	*√*	*√*	*√*	*√*	*√*	*√*	*√*	*√*	*√*	*√*	×	*√*
Download %	90	X	10	50	40	10	50	40	10	40	40	20	80	X	20
Upload %	50	X	50	30	20	50	30	20	50	20	10	70	30	X	70
Download Min (Kbps)	2000	X	500	2000	6000	500	2000	6000	500	1000	4000	200	1000	X	200
Upload Min (Kbps)	500	X	2000	500	500	2000	500	500	2000	1200	200	2000	1200	X	2000
Priority	3	X	1	3	2	1	3	2	1	1	3	2	1	X	2
Link bandwidth cap	100/100 Mbps			100/100 Mbps			10/5 Mbps			10/5 Mbps			10/5 Mbps		

**Table 4:** Calculation of max-rates in different scenarios.

	Scenario 1			Scenario 2			Scenario 3			Scenario 4			Scenario 5		
Slice	WEB	MM	SEC	WEB	MM	SEC	WEB	MM	SEC	WEB	MM	SEC	WEB	MM	SEC
Download Mbps	90	X	10	50	40	10	3	**6**	1	4	4	2	8	X	2
Upload Mbps	50	X	50	30	20	50	1.5	1	2.5	**1.2**	0.3	3.5	1.5	X	3.5

## Data Availability

The implementation of the proposed methodology is provided as open-source on the GitHub [54].

## Appendix A

As a practical example, we have presented, in Table A1, a smart vehicle application of multi-slice service bonding. Figure A1 presents a practical setup for such an application.

**Table A1:** The example of service grouping as slices for smart vehicle use case.

Slice	Services
**Navigation**	Update offline maps data Update point-of-interest database Current state of the road traffic Roadworks, incidents Weather Road conditions on a section of the road
**Parking**	Parking space availability Reservation of parking spaces
**Vehicle monitoring and safety**	The existence of a “black box” on the cloud Real-time vehicle location Vehicle video surveillance Remote control of locks and windows on the vehicle Real-time monitoring of all components and data transfer to the cloud (fuel sensor, battery condition, vehicle temperature, sensor operation check, etc.)
**Multimedia content**	Multimedia services (Multicast, VoD) Voice messaging
**Internet**	Content available on Internet services Video streaming services (YouTube, Netflix)
**Telephone**	Video conference Voice telephone
**Personal medical services**	Measurements of different health parameters and transfer information to the medical cloud
**Ad hoc communication between vehicles (V2V communication) ***	Abrupt braking information Road incident information (collision, pothole, slippage) Emergency communication Adjusting the light on the headlights Information on the need for extraordinary attention

* Note: V2V communication as ad hoc is independent of 6G telecom infrastructure service provider. However, some CarSDN air resources should be allocated (reserved) for this slice.
